# The Molecular Function and Clinical Role of Thyroid Stimulating Hormone Receptor in Cancer Cells

**DOI:** 10.3390/cells9071730

**Published:** 2020-07-20

**Authors:** Yu-De Chu, Chau-Ting Yeh

**Affiliations:** 1 Liver Research Center, Linkou Chang Gung Memorial Hospital, Taoyuan 333, Taiwan; yudechu19871003@gmail.com; 2 Liver Research Center, Chang Gung Memorial Hospital and Molecular Medicine Research Center, Chang Gung University, Taoyuan 333, Taiwan

**Keywords:** thyroid stimulating hormone receptor, cancer cells, extra-thyroid, G protein

## Abstract

The thyroid stimulating hormone (TSH) and its cognate receptor (TSHR) are of crucial importance for thyrocytes to proliferate and exert their functions. Although TSHR is predominantly expressed in thyrocytes, several studies have revealed that functional TSHR can also be detected in many extra-thyroid tissues, such as primary ovarian and hepatic tissues as well as their corresponding malignancies. Recent advances in cancer biology further raise the possibility of utilizing TSH and/or TSHR as a therapeutic target or as an informative index to predict treatment responses in cancer patients. The TSH/TSHR cascade has been considered a pivotal modulator for carcinogenesis and/or tumor progression in these cancers. TSHR belongs to a sub-group of family A G-protein-coupled receptors (GPCRs), which activate a bundle of well-defined signaling transduction pathways to enhance cell renewal in response to external stimuli. In this review, recent findings regarding the molecular basis of TSH/TSHR functions in either thyroid or extra-thyroid tissues and the potential of directly targeting TSHR as an anticancer strategy are summarized and discussed.

## 1. Introduction

Thyroid stimulating hormone receptor (TSHR) is a receptor for thyrostimulin and thyroid stimulating hormone (TSH, also called thyrotropin) [1]. TSHR is a cell surface glycoprotein receptor and belongs to the leucine-rich repeat (LGR) subfamily of G-protein-coupled receptors (GPCRs). There are two groups of receptors for gonadotrophic hormones: follitropin (FSH) and leutropin (LH)/choriogonadotropin (CG), that are structurally closely related to TSHR in humans and mammals [2]. Physiologically, upon binding of the blood circulating TSH on the surface of thyrocytes, TSHR is activated, switching on the coupled signaling pathways and thereby promoting the expression of downstream effector genes to control thyroid growth; thyrocyte differentiation; thyroid hormone synthesis, which includes thyroxine (T4) and triiodothyronine (T3); and hormone secretion [3]. The feedback loop composed of TSH–thyroid hormone has also been well characterized during the past decades [4]. Persistently low levels of thyroid hormone in the blood stream, or hypothyroidism, enhances TSH production and secretion, whereas over-production, or hyperthyroidism, represses this process in the hypothalamus. Notably, a similar regulatory loop does not exist between the expression levels of TSH and TSHR [5,6,7]. TSHR also serves as an autoantigen in Graves’ disease, where autoantibody binding leads to activation of the downstream signaling cascades, mimicking the effect of consistent stimulation of TSH and thus resulting in hyperthyroidism.

Expression of TSHR has been recognized in either benign or malignant thyrocytes, serving as a cognate receptor for TSH. Activation of the coupled signaling cascades through TSHR is considered the pivotal pathway for de novo carcinogenesis and/or tumor growth promoter for thyroid cancer. Clinically useful strategies have been proposed to treat well-differentiated thyroid cancer, which harbors a higher density of TSHR. For example, radio-iodine uptake into cancerous thyrocytes could be enhanced through transient elevation of the sodium-iodide symporter (NIS), a downstream gene of TSHR, by TSH stimulation [6]. This can also be achieved by either manipulating the thyroid hormone levels or exogenously supplementing the recombinant human TSH (rhTSH) [8]. Alternatively, activation of TSHR-mediated proliferative cues in malignant thyrocytes can be minimized therapeutically by inducing pharmacologic hyperthyroidism, leading to suppression of endogenous TSH. Recent investigation has unraveled novel possibilities to directly target TSHR in thyroid cancers by using selective small molecule antagonists of TSHR. Such strategies preclude the requirement of inducing systemic hyperthyroidism or the use of TSHR agonists to enhance the therapeutic index for drug delivery, such as radio-iodine uptake.

Although the roles remain elusive, the presence of TSHR in a bundle of extra-thyroid tissues [9], including some malignant tumors, such as ovarian cancer and hepatocellular carcinoma (HCC), has been found by researchers. Aroused interest in the roles of TSHR demands an inclusive review of evidences for its expression in distinct types of cancers. Herein, we review the reported evidence for the existence of TSHR in thyroid and non-thyroid tissues and its downstream coupled signaling as a mitogenic pathway in cancer cells. Additionally, we discuss potential new therapeutic strategies devised according to these findings.

## 2. Genomic Feature, Protein Structure and Distribution of TSHR

**Figure 1:**
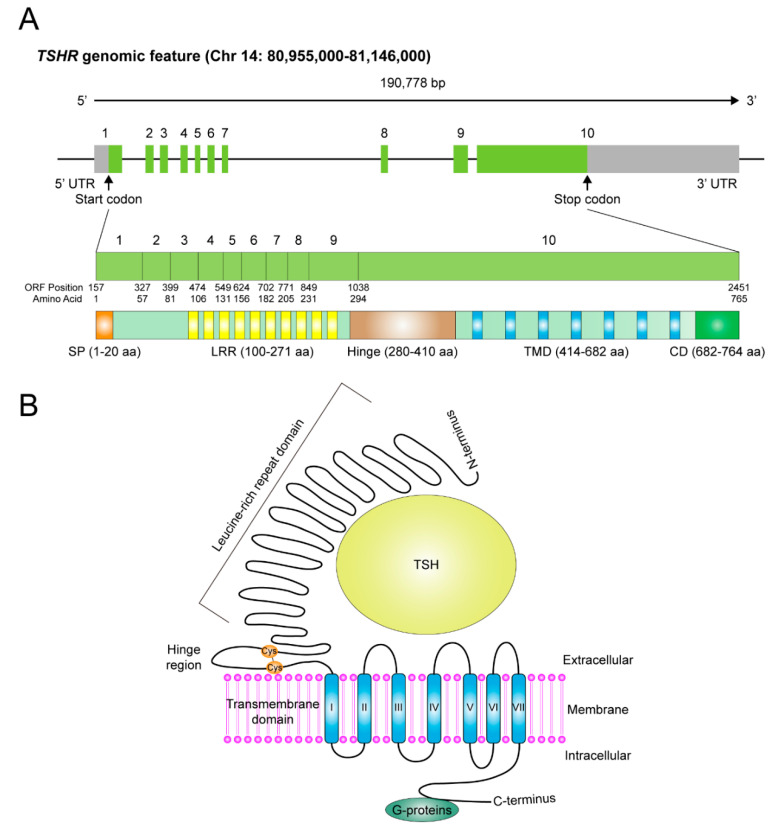
The genomic features and protein structure of thyroid stimulating hormone receptor (TSHR). (**A**) Schematic representation of the genomic structure of *TSHR* gene. The corresponding numbers of nucleotides and amino acids for the protein domains within the coding region are shown in this diagram. SP, signal peptide. LRR, leucine-rich repeat domain. TMD, transmembrane domain. CD, intracytoplasmic domain. (**B**) The schematic diagram represents the folded protein structure of TSHR, in which a large extracellular domain, including the hinge and leucine-rich repeat domain, the transmembrane domain and the intracytoplasmic domain are depicted. Cys, cysteine, where disulfide bonds formed.

The existence of TSHR was initially proposed and then confirmed during 1960–1980 by using thyroid tissue slices to demonstrate a bovine TSH (bTSH) binding surface protein [10,11]. However, it was not until the end of 1980s that the gene location of *TSHR*, together with its coding sequences, were identified and cloned from human chromosome 14q31 [12,13,14]. There are 10 exons in the *TSHR* gene. The former nine constitute the ectodomain (extracellular region) starting from the amino-terminus, whereas the tenth exon encodes seven transmembrane segments as well as a carboxyl-terminal region containing the intracytoplasmic domain (Figure 1A). The relationships have been established between genetic variations in *TSHR* gene and thyroid diseases, such as autoantibody-mediated and genetic variant-induced hyperactivation or repression of TSHR, causing hyper- or hypo-thyroidism. For instance, in patients with Graves’ disease or autoimmune-related hypothyroidism, such correlations have been extensively investigated and comprehensively reviewed during the past decades [15,16,17,18,19,20,21,22,23,24,25]. These topics are not included in this review.

The *TSHR* gene encodes a full-length protein of 764 amino acid residues, harboring a molecular weight of 87 kDa. Although it might exist as a single polypeptide chain under some specific situations in thyroid cells or in extra-thyroidal cells [26,27,28,29], most of the TSHR in thyroid cells are cleaved and divided into two subunits, A and B (or α and β)—A for an extracellular and B for a large intracellular portion, crosslinked by disulfide bonds, with exclusion of a 50-amino acid region (also called the hinge region), subjected to post-translational proteolysis [30,31,32,33,34,35] (Figure 1B). To achieve full functionality, TSHR also undergoes N-linked glycosylation, palmitoylation and other types of post-translational modifications [22,36,37]. The extracellular A-subunit possesses the TSH binding sites, composed of the leucine rich repeat domain (LRRD). Conformational change upon binding with TSH or stimulatory auto-antibodies leads to activation of TSHR and thereby switches on the intracellular B-subunit-coupled downstream signaling pathways [34,35].

It has been well studied and extensively reviewed that TSHR is distributed predominantly on the basolateral membrane of thyroid follicular cells [22]. In addition to thyroid tissues, the mRNA and protein expression of TSHR has also been identified in a bundle of other human and animal extra-thyroid tissues, including neural tissues, immune cells, ocular muscles, bone, adipocytes, erythrocytes, ovary and liver. The expression and functional role of TSHR in a variety of non-thyroid cancerous tissues, including melanoma, glioma, lung cancer, breast cancer, ovarian cancer and liver cancer, have been reported [38,39,40,41,42,43]. It is unfortunate that some of these findings are not confirmed by independent follow-up studies. As such, these findings will not be discussed in detail here. The evidence regarding *TSHR* mRNA and protein expression in extrathyroidal tissues, including normal and cancerous tissues, are summarized in Table 1.

## 3. The Signaling Pathways Downstream of TSHR in Response to Stimulatory Signals

TSHR belongs to a subfamily of family A G-protein-coupled seven-transmembrane receptors (GPCR) and activates canonical GPCR signaling cascades once stimulus signals are encountered [35,62]. Physiologically, the stimuli for TSHR activation on the surface of thyroid cells are of multiple distinct origins. Firstly, typical stimulation comes from binding of circulating TSH. Secondly, autoantibody-mediated binding mimicking that of TSH to the “pocket” of the extracellular region triggers TSHR activation. This is commonly observed in those with autoimmune disease, such as Graves’ disease. Thirdly, autonomous activation of TSHR caused by somatic or germline mutations in the *TSHR* gene, especially when the genetic variations are identified in sequences encoding the β (or B) subunit, as it directly associates with G proteins inside the cell membrane [35,63,64,65]. Another rarely discussed stimulating ligand for TSHR is an anciently-conserved hormone called thyrostimulin, a non-covalent heterodimeric hormone, also called orphan glycoprotein hormone or corticotroph-derived glycoprotein. It is composed of two protein subunits, glycoprotein hormone subunit alpha 2 (GPHA2) and glycoprotein hormone subunit beta 5 (GPHB5), identified initially from in vitro yeast-two hybrid and human cell-based experiments for its ability to physically interact with TSHR. These findings are further confirmed by colocalization experiments using tissues from the anterior pituitary of rats [66]. All of these stimulatory events contribute to the activation of signaling pathways downstream of TSHR-coupled G proteins.

**Figure 2:**
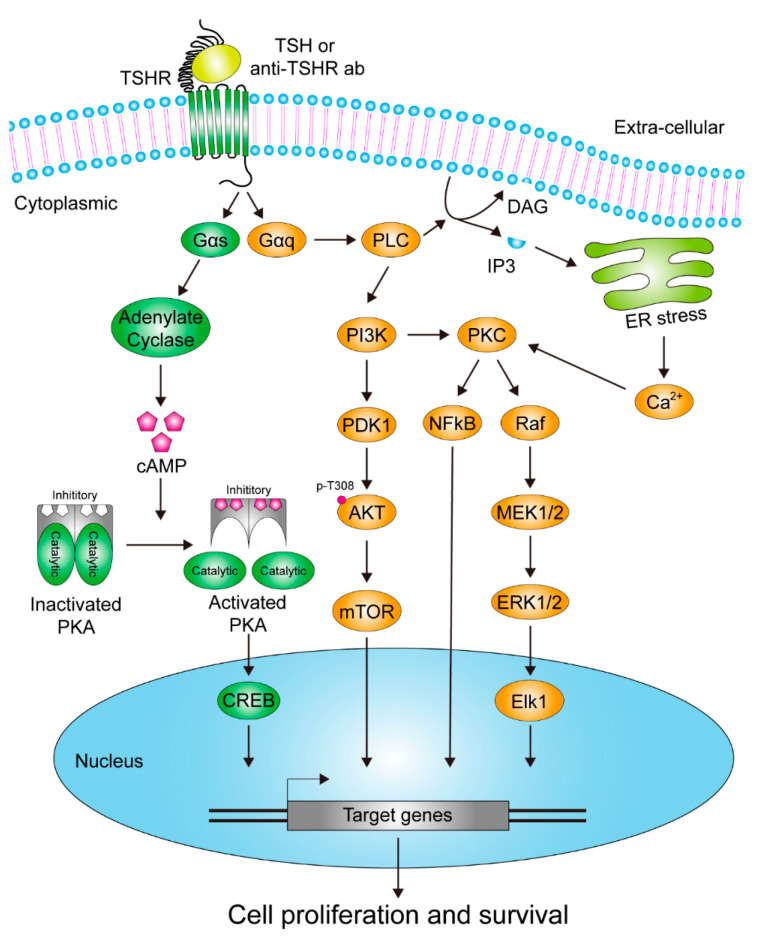
Diagrammatic representation of the identified signaling pathways activated by TSHR in thyrocytes.

Activation of TSHR and the linked signaling cascades through binding of circulating TSH or autoantibodies onto the surface of thyroid cells plays a pivotal role in controlling thyrocyte growth and in regulating thyroid hormone production/secretion [67,68]. This is executed through switching on different subtypes of G proteins and signaling pathways [69,70,71,72,73]. Among them, the Gαs- and Gαq-induced cascades are of the greatest importance [74,75,76,77], as they have been tightly linked to specific intracellular signal transductions downstream of TSHR in response to stimulations [78]. Generally, elevated activity of Gαs triggers adenylate cyclase (AC) activation, leading to generation of cyclic adenosine monophosphate (cAMP) to promote the pathways transduced by protein kinase A (PKA), also known as cAMP-dependent protein kinase, a complex consisting of two catalytic and two inhibitory subunits. Activation of PKA is achieved when binding of two cAMP molecules to each subunit occurs, resulting in conformation changes of the proteins and release of the inhibitory subunits. Among the proteins identified as potential substrates of PKA, direct phosphorylation of the cAMP-responsive element binding protein (CREB), a transcription factor, is the most frequently studied and reported. Upon CREB activation, expression of the downstream genes, most of them harboring the capability to enhance mitogenic effects, is then turned on in response to such stimulations [79,80,81,82,83,84,85,86] (Figure 2).

Another extensively investigated effect caused by activation of TSHR-coupled Gαq is a pathway in which TSHR/Gαq turns on phospholipase C (PLC) and/or phosphoinositide 3-kinase (PI3K) cascades so as to promote cell proliferation and survival. In this signaling pathway, elevated activity of PLC enhances decomposition of phosphatidylinositol 4,5-bisphosphate (PIP_2_) and thereby leads to release of inositol 1,4,5-trisphosphate (IP_3_) and generation of diacyl glycerol (DAG). As a result, protein kinase C (PKC), a calcium (Ca^2+^)-dependent protein kinase, is activated, so as to switch on downstream mitogen-activated protein kinase (MAPK) pathways. On the other hand, activation of PI3K-mediated signaling also triggers production of various 3-phosphorylated phosphoinositides, phosphatidylinositol (3,4,5)-trisphosphate (PI(3,4,5)P_3_), phosphatidylinositol 3-phosphate (PI3P) and phosphatidylinositol (3,4)-bisphosphate (PI(3,4)P_2_) for instance, to recruit and activate an assorted group of signaling proteins containing specific phosphoinositide-binding domains, such as the phosphoinositide-dependent kinase-1 (PDK1) and pleckstrin homology domains (PH domains) harboring protein AKT. The activated PDK1 then phosphorylates AKT at the T308 residue to partially elevate its function, although exercising the full functionality of AKT needs additional phosphorylation at S473 [69,70,87,88,89] (Figure 2).

A similar mechanism seems to be employed by the ancestrally-conserved thyrostimulin, which is estimated to have 30-fold stronger binding affinity to TSHR than TSH, acting as a circulatory hormone released from the anterior pituitary or a paracrine hormone secreted from neighboring tissues [59,90,91]. Thyrostimulin binds to the binding sites on TSHR, where GPHA2 binds to the transmembrane domain and GPHB5 associates with the extracellular domain, leading to activation of TSHR-mediated signaling cascades. The binding between GPHA2 and TSHR has been found to be similar to that between TSH and TSHR, wherein the N-linked glycosylation at specific residues either on GPHA2 or TSH is pivotal for activation of TSHR [92,93]. Although the existence of thyrostimulin in humans remains doubtful currently, its roles and functions have been extensively investigated in a bundle of animal models (for a comprehensive review of thyrostimulin, see [90]).

## 4. TSHR in Thyroid Cancer Cells

Expression of TSHR is predominantly identified in thyroidal tissues, either in benign and malignant thyroid tissues, serving as a target mainly for TSH [9]. The TSHR-mediated growth of thyroid was initially observed in TSH-secreting pituitary adenoma and Graves’ disease [94,95]. Subsequently, multiple large cohort studies have proposed that higher serum TSH levels are linked to an increased risk of thyroid cancer [96]. The activated TSHR-induced signaling cascades, as mentioned above, have been shown to serve as oncogenic pathways in thyroid cancer, especially in tumors carrying mutations at V600E in B-Raf proto-oncogene. Furthermore, it has been demonstrated by subsequent studies that TSH signaling overcomes senescence induced by *BRAF*^V600E^ mutations in vitro in cell culture and in vivo in mice [97,98,99,100]. Moreover, by using a TSHR-knockout mouse model (TSHR(-/-)) to study the role of TSH/TSHR-mediated growth signaling, it was discovered that existing additional oncogenic mutation, such as that in TRbeta(PV/PV) mice, is indispensable for follicular thyroid cancer (FTC) cells to metastasize [101], indicating that although the signaling transduction downstream of TSH/TSHR plays an important role in modulating thyroidal cell proliferation, other coexisting mutations capable of turning on other signaling cascades are required to establish an invasive phenotype. Except for the TSH-induced activation of TSHR, there are numerous spontaneously occurring mutations located within the *TSHR* gene, which are identified in thyroid cancer patients. These can be either germline or tumor-specific mutations, carrying the ability to automatously turn on downstream cascades and thereby promoting cell proliferation, such as V509A, C672Y and M453T [65,102]. Taken together, TSHR has a growth-promoting or oncogene-like function in thyroid cancer, although other co-existing mutations are required to establish a fully malignant phenotype. Nevertheless, clinically, the expression levels of TSHR seem to be downregulated in patients with more advanced stages of thyroid cancer, especially for those with poorly differentiated cell types, suggesting the existence of an uncharacterized selection/adaption mechanism [103].

## 5. TSHR in Extra-Thyroid Cancer Cells

### 5.1. Ovarian Cancer

**Figure 3:**
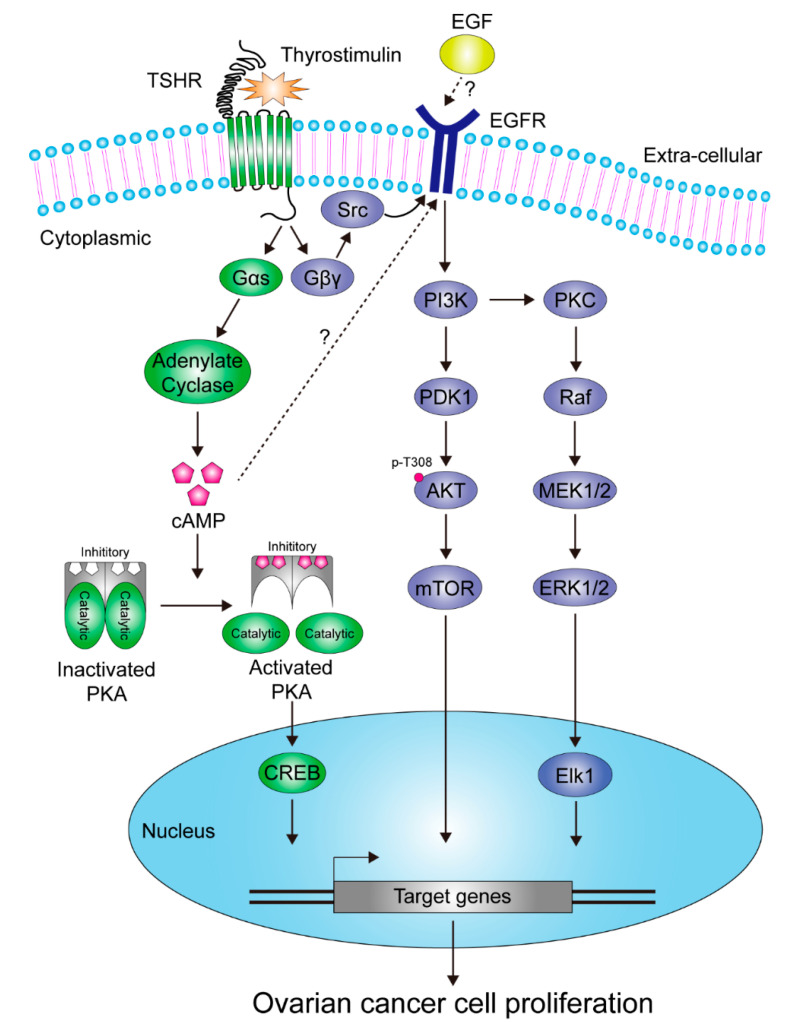
Schematic representation of the identified effectors and pathways downstream of TSHR in response to thyrostimulin stimulation in ovarian cancer cells.

Existence of both TSHR and thyroid hormone receptor (TRs) in human ovarian tissue was initially reported in 2009 by Aghajanova et al., using immunohistochemical staining and reverse transcription polymerase chain reaction (RT-PCR). Functionality of TSHR and TRs in primary human ovarian tissues under TSH or thyroid hormone stimulation was also demonstrated through assessing downstream signaling pathways or metabolites [58]. Subsequent studies were in agreement with this, finding that TSHR was indeed expressed in ovarian tissues, and furthermore in cancerous cells [42,59,61]. Among these studies, Sun et al. confirmed that expression of *TSHR* mRNA not only occurs in ovaries but also in the oviduct by use of RT-PCR assay in either animal models or human ovarian cell lines. Full functionality of TSHR in NIH:OVCR-3 ovarian cancer cells was also demonstrated. Moreover, the expression of *TSHR* was periodically up-regulated by the gonadotropin-driven cAMP cascade and down-regulated by estradiol production in rat ovaries. Intriguingly, contradictory to Aghajanova’s findings (although both may be true), they demonstrated that it was not TSH but thyrostimulin which acted as a paracrine regulator to modulate activation of TSHR in mammalian ovaries [59]. Recently, they also discovered that the paracrine thyrostimulin-mediated activation of TSHR switched on canonical G-protein coupled signaling pathways and trans-regulated activation of epithelial growth factor receptor (EGFR) to promote ovarian cancer cell proliferation [61] (Figure 3).

The results reported by Huang et al. indicated that TSHR served as a master oncogenic protein in response to thyrostimulin stimulation in ovarian cancer. This concept is partially confirmed by another publication showing that *TSHR* mRNA expression level could serve as a biomarker to predict the response of adjuvant intravenous platinum-taxane chemotherapy in patients with ovarian cancer, wherein those with increased *TSHR* mRNA level are associated with poorer clinical outcomes [60]. These lines of evidence partially uncover the growth-promoting role of TSH/TSHR and/or thyrostimulin/TSHR in ovary and ovarian cancer. Nevertheless, the details of underlying mechanisms by which TSHR modulates oncogenesis or tumor growth of ovarian cancer, and new strategies designed accordingly to intervene these processes, still await further exploration.

### 5.2. Liver Cancer

The presence of TSHR on the cell surface of human liver tissues as well as a hepatocyte cell line (L-02) was first demonstrated by Zhang et al. via RT-PCR and immuno-fluorescence assay (IFA), respectively [51]. The functionality of TSHR, driving cAMP accumulation in response to the stimulation of bovine TSH (bTSH) and immunoglobulins isolated from patients with Graves’ disease, was also demonstrated in this study. Subsequently, these findings were confirmed in an animal model by showing that TSH/TSHR played a role in maintaining hepatic blood glucose, triacylglycerol and bile acid homeostasis [104,105,106]. Lines of evidence from other studies also indicated that TSH/TSHR modulated progression of liver-associated metabolic diseases in human and in mice model [52,106,107,108,109,110]. Recently, it has been unveiled that abnormality in metabolic processes can lead to enhanced carcinogenesis and/or metastasis of several cancers, including liver cancer [111]. However, it was not until 2018 that Shih and coworkers demonstrated that the levels of TSHR predicted clinical outcomes of patients with hepatocellular carcinoma (HCC), of which poorer survival rates were observed in those with higher TSHR expression, and the presence of TSH altered sensitivity of HCC cells to cisplatin, a chemotherapeutic drug for HCC, in vitro [43]. Moreover, several mutations within exon 10 of the *TSHR* gene were identified in HCC tissues, despite the fact that the roles of these mutants in promoting tumorigenesis or cancer progression in HCC remained uncharacterized.

In liver cancer, another frequently debated issue is the roles of the thyroid hormones T3 and T4 in tumorigenesis, cell growth and metastasis. Thyroid hormones might impact HCC growth through various pathways. According to current understandings in this field, most of the literature has considered T3/T4 a potential tumor suppressor, while others have considered them to be oncogenic promoters in HCC (for a comprehensive review of the effects of thyroid hormones in HCC, see [112]). Some of these studies provided a possible explanation for the finding that higher TSH and free T4 levels in blood were associated with poorer prognoses and larger tumor size in unresectable HCC patients [113]. Intriguingly, another independent study showed that higher TSH or free T4 concentrations in blood were associated with better clinical outcomes in HCC patients receiving chemotherapy, but not sorafenib (a targeted anticancer drug) treatment [114]. According to this study, it might be better to treat HCC patients with higher TSH and free T4 levels by chemotherapy instead of the targeted drug sorafenib, although the mechanisms behind these observations still require future elucidation. Overall, these results partly unveil the role and applicability of TSH/T4 in liver cancer treatment. Notably, the oncogenic role of thyroid hormones (not TSH/TSHR) in HCC is still under debate [115,116].

## 6. Clinical Usefulness of TSHR Related Pathways in Anticancer Therapies

For thyroid cancer, there are lines of evidence revealing a strong association between high levels of T4 or T3 and reduced thyroid cancer growth, as thyroid hormones suppress TSH secretion from the pituitary gland [117]. Subsequently, by using levothyroxine, an artificially synthesized mimic of T4, to treat patients, the sizes of thyroid nodules are reduced and metastases of thyroid cancer are restrained [118,119,120,121]. Indirect TSH suppression by exogenous levothyroxine administration remains a mainstay of clinical management for differentiated thyroid cancer nowadays, especially for patients under high risk of recurrence [121]. However, a more individualized treatment strategy regarding TSH suppression has to be adopted, because it is of little benefit for patients with low risk of recurrence and there is an increased risk of bone loss and cardiovascular diseases, owing to adverse effects caused by exogenous subclinical hyperthyroidism [122,123]. On the other hand, the TSHR-mediated upregulation of sodium-iodide symporter expression is frequently used to enhance the radioiodine uptake in ablative radioiodine therapy. This can be achieved by means of either endogenous TSH stimulation through thyroxine withdrawal or exogeneous administration of recombinant human TSH [8]. Additionally, evidence from clinical studies shows that expression levels of TSHR serve as a marker to predict the efficacy of these therapies [124,125].

Several small molecule kinase inhibitors, identified as thyroid cancer cell proliferation repressors, rather than as tumoricidal, have been used as the first-line strategy to treat progressive radioiodine-resistant differentiated thyroid cancer. They can provide prolonged progression-free survival but not a cure [8]. Moreover, the toxicities of this class of agents hinder its widespread use. A combination or sequential treatment strategy to apply the TSHR-mediated pathway manipulation and kinase inhibitors may be considered in the future.

In order to improve the therapeutic strategies for cancer, the term “theranostics” has been created. Owing to the dedication of scientists in this field, several novel strategies and methods to deliver drugs have been established to minimize the off-target/mis-target effects and maximize the proper delivery of drugs to where they are needed. These include several attractive models of nanoliposomes, organ-specific targeting and immune system evasion methods, which can be achieved by molecules embedded in the lipid bilayer. To target the TSH/TSHR pathway, the nanoliposomes coated with fragments of TSH were used to compete with the binding of TSHR in vitro and in vivo [126]. In this study, the researchers showed that the nanoliposomes packed with gemcitabine, a chemotherapeutic agent, exhibited higher efficacy against the proliferation of thyroid cancer cell line FTC-133 in vitro than uncoating TSH liposomal gemcitabine and free gemcitabine. A similar study confirmed such notions by packing cisplatin [127]. These studies indeed provide a novel strategy, taking advantage of the TSH/TSHR pathway to treat thyroid cancer. Inconvenience exists, however, as liposomes need to be used freshly and are unstable under long-term storage, raising the needs of an alternative or improved design.

Pharmacologic suppression of endogenous TSH via the negative feedback loop for the purpose of inhibiting TSHR-mediated growth signaling often leads to subclinical hyperthyroidism. Considering the stability and easily manufactured nature of protein or peptidyl drugs, it may be more reasonable to develop antagonists of TSHR to avoid systemic side effects, while manifesting effectiveness to inhibit thyroid cancer growth. Such small-molecule antagonists of TSHR attract much attention from scientists who are dedicated to developing new treatments for Graves’ disease, a pathological condition caused by binding of stimulatory autoantibodies to TSHR to induce hyperthyroidism. However, unsatisfactory results remain, as most molecules cannot achieve sufficient specificity in vivo to substantially inhibit TSHR signaling, although several synthesized compounds have been generated that are capable of suppressing TSHR expression or its downstream signaling to a certain extent [128]. These compounds, including antagonists and inverse agonists, are of minimal/mild effectiveness in suppressing TSHR signal transduction, as only around 50% and 39% inhibitory effects for cAMP production could be achieved when stimulated by TSH or sera derived from patients with Graves’ disease, respectively [129,130]. Recently, a novel small molecule antagonist of TSHR has been identified by drug screening. The researchers claimed that a selective inhibitory effect could be observed in TSHR but not in those closely related receptors, such as follitropin and lutropin receptors [131]. Indeed, it would be very helpful if an oral small molecule drug can be approved for clinical use to treat patients with thyroid cancer or Graves’ disease, but there remains a long way to go, as the present findings are still limited to cell-based assays and mouse models, rather than clinical trials.

On the other hand, it has not been explored whether patients with extra-thyroid cancers can be treated by targeting TSHR or the related pathways. Only a few studies have demonstrated such potentiality in ovarian and liver cancers [43,60,114]. Directly targeting TSHR in extra-thyroid cancer seems to be impractical, as the systemic effect caused by such treatment is difficult to avoid. TSHR expression is most abundant in thyroid cells, compared to all other extra-thyroid tissues or organs. As such, unless a specific liver or ovarian cancer delivering or targeting system can be developed, the TSHR-targeting strategy cannot be applied to extra-thyroid cancers. However, by use of the index obtained from the levels of TSH and free T4, one can select a better strategy to treat patients with HCC [113,114].

## 7. Conclusions

The molecular function and clinical role of TSHR have been extensively explored in both benign and malignant thyroid tissues. Accordingly, it has been proposed that TSHR might serve as a candidate target for anticancer therapy [103]. Our current understandings of the anticancer role of TSHR in thyroid cancer come mostly from the effectiveness of the therapeutic strategies manipulating the TSH/TSHR signaling pathways, for example, by administration of levothyroxine. Such strategies remain largely unchanged during the past decades.

In addition to thyroid cancer, the aforementioned strategies targeting the TSH/TSHR pathways can potentially be used to fight against extra-thyroid cancers, provided that the malignant tissues harbor higher expression levels of TSHR on the surface of cancerous cells. To date, expression of TSHR mRNA and protein have been detected in a variety of tissues and extra-thyroid cancers, although our knowledge regarding the functional characteristics of these extra-thyroid TSHR remains limited. Several difficulties have to be overcome, however, prior to a possible clinical application. For example, the expression levels of TSHR in the extra-thyroid cancers have to be high enough that the alteration of TSHR function induced by the cognate ligands is great enough to elicit the desired anticancer responses. It is highly likely that the TSHR expression can vary not only cancer-to-cancer but also patient-to-patient.

In the era of precision medicine and targeted therapy, TSHR has been proposed to be a potent target against thyroid cancer with several experimental compounds under development. This potential is supported by the success of the therapeutic strategies to manipulate TSH/TSHR function in treating differentiated thyroid cancer. Although there is hope for targeting TSHR in the treatment of extra-thyroid cancers, much work needs to be done before this becomes a clinical reality.

## Figures and Tables

**Table 1:** Summary of evidences of TSHR expression in human extra-thyroid tissues or cells.

Benign Tissues	Expression ^a^	Functionality ^b^	Reference
Adipose tissue	Both mRNA and protein	Yes	[44,45,46]
Adrenal	Both mRNA and protein	No	[47]
Endometrium	Both mRNA and protein	No	[48]
Erythrocytes	Protein	Yes	[49,50]
Kidney	Both mRNA and protein	No	[47]
Liver	Both mRNA and protein	Yes	[51,52]
Lymphocytes	Both mRNA and protein	No	[53]
Pituitary	Both mRNA and protein	No	[54]
Cardiac muscle	mRNA	Yes	[55]
Hair follicles	Both mRNA and protein	Yes	[56]
Vascular smooth muscle	Both mRNA and protein	Yes	[57]
Ovary	Both mRNA and protein	Yes	[58,59]
**Malignant tissues**	**Expression ^a^**	**Functionality ^b^**	**Reference**
Melanoma	Both mRNA and protein	Yes	[39]
Glioma	mRNA	No	[38]
Lung cancer	Protein	No	[40]
Breast cancer	Both mRNA and protein	No	[41]
Ovarian cancer	Both mRNA and protein	Yes	[42,59,60,61]
Hepatocellular carcinoma	Protein	Yes	[43]

^a^ Detection of TSHR mRNA was performed by RT-PCR or real-time quantitative-PCR, whereas detection of TSHR protein was conducted by immunohistochemical staining or Western blot. ^b^ Functionality examination was assayed by quantitating cAMP production or determining downstream effectors activation.

## References

[1] Neumann S., Raaka B.M., Gershengorn M.C. (2009). Human TSH receptor ligands as pharmacological probes with potential clinical application. Expert Rev. Endocrinol. Metab..

[2] Troppmann B., Kleinau G., Krause G., Gromoll J. (2013). Structural and functional plasticity of the luteinizing hormone/choriogonadotrophin receptor. Hum. Reprod Update.

[3] Persani L., Gelmini G., Marelli F., Beck-Peccoz P., Bonomi M. (2011). Syndromes of resistance to TSH. Ann. Endocrinol..

[4] Schroeder A.C., Privalsky M.L. (2014). Thyroid hormones, t3 and t4, in the brain. Front. Endocrinol..

[5] Maenhaut C., Brabant G., Vassart G., Dumont J.E. (1992). In vitro and in vivo regulation of thyrotropin receptor mRNA levels in dog and human thyroid cells. J. Biol. Chem..

[6] Bruno R., Ferretti E., Tosi E., Arturi F., Giannasio P., Mattei T., Scipioni A., Presta I., Morisi R., Gulino A. (2005). Modulation of thyroid-specific gene expression in normal and nodular human thyroid tissues from adults: An in vivo effect of thyrotropin. J. Clin. Endocrinol. Metab..

[7] Schuppert F., Deiters S., Rambusch E., Sierralta W., Dralle H., Mühlen A.V.Z. (1996). TSH-receptor expression and human thyroid disease: Relation to clinical, endocrine, and molecular thyroid parameters. Thyroid.

[8] Haugen B.R., Alexander E.K., Bible K.C., Doherty G.M., Mandel S.J., Nikiforov Y.E., Pacini F., Randolph G.W., Sawka A.M., Schlumberger M. (2016). 2015 American Thyroid Association Management Guidelines for Adult Patients with Thyroid Nodules and Differentiated Thyroid Cancer: The American Thyroid Association Guidelines Task Force on Thyroid Nodules and Differentiated Thyroid Cancer. Thyroid.

[9] Williams G.R. (2011). Extrathyroidal expression of TSH receptor. Ann. Endocrinol.

[10] Pastan I., Roth J., Macchia V. (1966). Binding of hormone to tissue: The first step in polypeptide hormone action. Proc. Natl. Acad. Sci. USA.

[11] Amir S.M., Carraway T.F., Kohn L.D., Winand R.J. (1973). The Binding of Thyrotropin to Isolated Bovine Thyroid Plasma Membranes. J. Biol. Chem..

[12] Parmentier M., Libert F., Maenhaut C., Lefort A., Gerard C., Perret J., Sande J.V., Dumont J., Vassart G. (1989). Molecular cloning of the thyrotropin receptor. Science.

[13] Nagayama Y., Kaufman K.D., Seto P., Rapoport B. (1989). Molecular cloning, sequence and functional expression of the cDNA for the human thyrotropin receptor. Biochem. Biophys. Res. Commun..

[14] Misrahi M., Loosfelt H., Atger M., Sar S., Guiochon-Mantel A., Milgrom E. (1990). Cloning, sequencing and expression of human TSH receptor. Biochem. Biophys. Res. Commun..

[15] Akamizu T. (2001). Antithyrotropin Receptor Antibody: An Update. Thyroid.

[16] Bahn R.S. (2012). Autoimmunity and Graves’ disease. Clin. Pharm..

[17] Bahn R.S., Dutton C.M., Natt N., Joba W., Spitzweg C., Heufelder A.E. (1998). Thyrotropin receptor expression in Graves’ orbital adipose/connective tissues: Potential autoantigen in Graves’ ophthalmopathy. J. Clin. Endocrinol. Metab..

[18] Davies T.F. (2005). Thyrotropin receptor-associated diseases: From adenomata to Graves disease. J. Clin. Investig..

[19] Kohn L.D., Harii N. (2003). Thyrotropin receptor autoantibodies (TSHRAbs): Epitopes, origins and clinical significance. Autoimmunity.

[20] Ludgate M. (2000). Animal models of Graves’ disease. Eur. J. Endocrinol..

[21] Michalek K., Morshed S.A., Latif R., Davies T.F. (2009). TSH receptor autoantibodies. Autoimmun. Rev..

[22] Rapoport B., Chazenbalk G.D., Jaume J.C., Mclachlan S.M. (1998). The thyrotropin (TSH) receptor: Interaction with TSH and autoantibodies. Endocr. Rev..

[23] Rapoport B., McLachlan S.M. (2007). The Thyrotropin Receptor in Graves’ Disease. Thyroid.

[24] Diana T., Olivo P.D., Kahaly G.J. (2018). Thyrotropin Receptor Blocking Antibodies. Horm. Metab. Res..

[25] Davies T.F., Yin X., Latif R. (2010). The genetics of the thyroid stimulating hormone receptor: History and relevance. Thyroid.

[26] Russo D., Chazenbalk G.D., Nagayama Y., Wadsworth H.L., Seto P., Rapoport B. (1991). A new structural model for the thyrotropin (TSH) receptor, as determined by covalent cross-linking of TSH to the recombinant receptor in intact cells: Evidence for a single polypeptide chain. Mol. Endocrinol..

[27] Endo T., Ikeda M., Ohmori M., Anzai E., Haraguchi K., Onaya T. (1992). Single subunit structure of the human thyrotropin receptor. Biochem. Biophys. Res. Commun..

[28] Chazenbalk G.D., Kakinuma A., Jaume J.C., Mclachlan S.M., Rapoport B. (1996). Evidence for negative cooperativity among human thyrotropin receptors overexpressed in mammalian cells. Endocrinology.

[29] Chen C.-R., Chazenbalk G.D., Wawrowsky K.A., McLachlan S.M., Rapoport B. (2006). Evidence that human thyroid cells express uncleaved, single-chain thyrotropin receptors on their surface. Endocrinology.

[30] Buckland P.R., Howells R.D., Rickards C.R., Smith B.R. (1985). Affinity-labelling of the thyrotropin receptor. Characterization of the photoactive ligand. Biochem. J..

[31] Kajita Y., Rickards C.R., Buckland P.R., Howells R.D., Smith B.R. (1985). Analysis of thyrotropin receptors by photoaffinity labelling. Orientation of receptor subunits in the cell membrane. Biochem. J..

[32] Buckland P.R., Strickland T.W., Pierce J.G., Smith B.R. (1985). TSH crosslinks to the TSH receptor through the beta subunit. Endocrinology.

[33] Loosfelt H., Pichon C., Jolivet A., Misrahi M., Caillou B., Jamous M., Vannier B., Milgrom E. (1992). Two-subunit structure of the human thyrotropin receptor. Proc. Natl. Acad. Sci. USA.

[34] Rapoport B., McLachlan S.M. (2016). TSH Receptor Cleavage into Subunits and Shedding of the A-Subunit; A Molecular and Clinical Perspective. Endocr. Rev..

[35] Kleinau G., Worth C.L., Kreuchwig A., Biebermann H., Marcinkowski P., Scheerer P., Krause G. (2017). Structural-Functional Features of the Thyrotropin Receptor: A Class A G-Protein-Coupled Receptor at Work. Front. Endocrinol..

[36] Kursawe R., Paschke R. (2007). Modulation of TSHR signaling by posttranslational modifications. Trends Endocrinol. Metab..

[37] Zabczynska M., Kozlowska K., Pochec E. (2018). Glycosylation in the Thyroid Gland: Vital Aspects of Glycoprotein Function in Thyrocyte Physiology and Thyroid Disorders. Int. J. Mol. Sci..

[38] Vastrad B., Vastrad C., Godavarthi A., Chandrashekar R. (2017). Molecular mechanisms underlying gliomas and glioblastoma pathogenesis revealed by bioinformatics analysis of microarray data. Med. Oncol..

[39] Ellerhorst J.A., Sendi-Naderi A., Johnson M.K., Cooke C.P., Dang S.M., Diwan A.H. (2006). Human melanoma cells express functional receptors for thyroid-stimulating hormone. Endocr. Relat. Cancer.

[40] Kim J.W., Lee S., Lui N., Choi H., Mulvihill M., Fang L.T., Kang H.C., Kwon Y.W., Jablons D., Kim I.J. (2012). A somatic TSHR mutation in a patient with lung adenocarcinoma with bronchioloalveolar carcinoma, coronary artery disease and severe chronic obstructive pulmonary disease. Oncol. Rep..

[41] Govindaraj V., Yaduvanshi N.S., Krishnamachar H., Rao A.J. (2012). Expression of thyroid-stimulating hormone receptor, octamer-binding transcription factor 4, and intracisternal A particle-promoted polypeptide in human breast cancer tissues. Horm. Mol. Biol. Clin. Investig.

[42] Gyftaki R., Liacos C., Politi E., Liontos M., Saltiki K., Papageorgiou T., Thomakos N., Haidopoulos D., Rodolakis A., Alevizaki M. (2014). Differential transcriptional and protein expression of thyroid-stimulating hormone receptor in ovarian carcinomas. Int. J. Gynecol. Cancer.

[43] Shih Y.L., Huang Y.H., Lin K.H., Chu Y.D., Yeh C.T. (2018). Identification of Functional Thyroid Stimulating Hormone Receptor and TSHR Gene Mutations in Hepatocellular Carcinoma. Anticancer Res..

[44] Janson A., Rawet H., Perbeck L., Marcus C. (1998). Presence of thyrotropin receptor in infant adipocytes. Pediatr. Res..

[45] Bell A., Gagnon A., Grunder L., Parikh S.J., Smith T.J., Sorisky A. (2000). Functional TSH receptor in human abdominal preadipocytes and orbital fibroblasts. Am. J. Physiol. Cell Physiol..

[46] Murakami M., Kamiya Y., Morimura T., Araki O., Imamura M., Ogiwara T., Mizuma H., Mori M. (2001). Thyrotropin receptors in brown adipose tissue: Thyrotropin stimulates type II iodothyronine deiodinase and uncoupling protein-1 in brown adipocytes. Endocrinology.

[47] Dutton C.M., Joba W., Spitzweg C., Heufelder A.E., Bahn R.S. (1997). Thyrotropin receptor expression in adrenal, kidney, and thymus. Thyroid.

[48] Aghajanova L., Stavreus-Evers A., Lindeberg M., Landgren B.M., Sparre L.S., Hovatta O. (2011). Thyroid-stimulating hormone receptor and thyroid hormone receptors are involved in human endometrial physiology. Fertil. Steril..

[49] Balzan S., Nicolini G., Forini F., Boni G., Del Carratore R., Nicolini A., Carpi A., Iervasi G. (2007). Presence of a functional TSH receptor on human erythrocytes. Biomed. Pharm..

[50] Balzan S., Carratore R.D., Nicolini G., Forini F., Lubrano V., Simili M., Benedetti P.A., Iervasi G. (2009). TSH induces co-localization of TSH receptor and Na/K-ATPase in human erythrocytes. Cell Biochem. Funct..

[51] Zhang W., Tian L.M., Han Y., Ma H.Y., Wang L.C., Guo J., Gao L., Zhao J.J. (2009). Presence of thyrotropin receptor in hepatocytes: Not a case of illegitimate transcription. J. Cell. Mol. Med..

[52] Li Y., Wang L., Zhou L., Song Y., Ma S., Yu C., Zhao J., Xu C., Gao L. (2017). Thyroid stimulating hormone increases hepatic gluconeogenesis via CRTC2. Mol. Cell Endocrinol..

[53] Coutelier J.-P., Kehrl J.H., Bellur S.S., Kohn L.D., Notkins A.L., Prabhakar B.S. (1990). Binding and functional effects of thyroid stimulating hormone on human immune cells. J. Clin. Immunol..

[54] Prummel M.F., Brokken L.J.S., Meduri G., Misrahi M., Bakker O., Wiersinga W.M. (2000). Expression of the thyroid-stimulating hormone receptor in the folliculo-stellate cells of the human anterior pituitary. J. Clin. Endocrinol. Metab..

[55] Drvota V., Janson A., Norman C., Sylven C., Haggblad J., Bronnegard M., Marcus C. (1995). Evidence for the presence of functional thyrotropin receptor in cardiac muscle. Biochem. Biophys. Res. Commun..

[56] Bodo E., Kromminga A., Biro T., Borbiro I., Gaspar E., Zmijewski M.A., van Beek N., Langbein L., Slominski A.T., Paus R. (2009). Human female hair follicles are a direct, nonclassical target for thyroid-stimulating hormone. J. Invest. Derm..

[57] Tian L., Ni J., Guo T., Liu J., Dang Y., Guo Q., Zhang L. (2014). TSH stimulates the proliferation of vascular smooth muscle cells. Endocrine.

[58] Aghajanova L., Lindeberg M., Carlsson I.B., Stavreus-Evers A., Zhang P., Scott J.E., Hovatta O., Skjöldebrand-Sparre L. (2009). Receptors for thyroid-stimulating hormone and thyroid hormones in human ovarian tissue. Reprod. Biomed. Online.

[59] Sun S.C., Hsu P.J., Wu F.J., Li S.H., Lu C.H., Luo C.W. (2010). Thyrostimulin, but not thyroid-stimulating hormone (TSH), acts as a paracrine regulator to activate the TSH receptor in mammalian ovary. J. Biol. Chem..

[60] Seagle B.L., Eng K.H., Yeh J.Y., Dandapani M., Schiller E., Samuelson R., Odunsi K., Shahabi S. (2016). Discovery of candidate tumor biomarkers for treatment with intraperitoneal chemotherapy for ovarian cancer. Sci. Rep..

[61] Huang W.L., Li Z., Lin T.Y., Wang S.W., Wu F.J., Luo C.W. (2016). Thyrostimulin-TSHR signaling promotes the proliferation of NIH:OVCAR-3 ovarian cancer cells via trans-regulation of the EGFR pathway. Sci. Rep..

[62] Vassart G., Pardo L., Costagliola S. (2004). A molecular dissection of the glycoprotein hormone receptors. Trends Biochem. Sci..

[63] Tuncel M. (2017). Thyroid Stimulating Hormone Receptor. Mol. Imaging. Radionucl..

[64] Kleinau G., Neumann S., Grüters A., Krude H., Biebermann H. (2013). Novel Insights on Thyroid-Stimulating Hormone Receptor Signal Transduction. Endocr. Rev..

[65] Duprez L., Parma J., Sande J.V., Allgeier A., Leclère J., Schvartz C., Delisle M.-J., Decoulx M., Orgiazzi J., Dumont J. (1994). Germline mutations in the thyrotropin receptor gene cause non-autoimmune autosomal dominant hyperthyroidism. Nat. Genet..

[66] Hsu S.Y., Nakabayashi K., Bhalla A. (2002). Evolution of Glycoprotein Hormone Subunit Genes in Bilateral Metazoa: Identification of Two Novel Human Glycoprotein Hormone Subunit Family Genes, GPA2 and GPB5. Mol. Endocrinol..

[67] Postiglione M.P., Parlato R., Rodriguez-Mallon A., Rosica A., Mithbaokar P., Maresca M., Marians R.C., Davies T.F., Zannini M.S., De Felice M. (2002). Role of the thyroid-stimulating hormone receptor signaling in development and differentiation of the thyroid gland. Proc. Natl. Acad. Sci. USA.

[68] Vassart G., Dumont J.E. (1992). The Thyrotropin Receptor and the Regulation of Thyrocyte Function and Growth. Endocr. Rev..

[69] Allgeier A., Offermanns S., Sande J.V., Spicher K., Schultz G., Dumont J. (1994). The human thyrotropin receptor activates G-proteins Gs and Gq/11. J. Biol. Chem..

[70] Laugwitz K.-L., Allgeier A., Offermanns S., Spicher K., Sande J.V., Dumont J.E., Schultz G. (1996). The human thyrotropin receptor: A heptahelical receptor capable of stimulating members of all four G protein families. Proc. Natl. Acad. Sci. USA.

[71] Sande J.V., Raspé E., Perret J., Lejeune C., Maenhaut C., Vassart G., Dumont J.E. (1990). Thyrotropin activates both the cyclic AMP and the PIP2 cascades in CHO cells expressing the human cDNA of TSH receptor. Mol. Cell Endocrinol..

[72] Buch T.R., Biebermann H., Kalwa H., Pinkenburg O., Hager D., Barth H., Aktories K., Breit A., Gudermann T. (2008). G13-dependent activation of MAPK by thyrotropin. J. Biol. Chem..

[73] Krause K., Boisnard A., Ihling C., Ludgate M., Eszlinger M., Krohn K., Sinz A., Fuhrer D. (2012). Comparative proteomic analysis to dissect differences in signal transduction in activating TSH receptor mutations in the thyroid. Int. J. Biochem. Cell Biol..

[74] Kero J., Ahmed K., Wettschureck N., Tunaru S., Wintermantel T., Greiner E., Schutz G., Offermanns S. (2007). Thyrocyte-specific Gq/G11 deficiency impairs thyroid function and prevents goiter development. J. Clin. Invest..

[75] Ledent C., Parmentier M., Maenhaut C., Taton M., Pirson I., Lamy F., Roger P., Dumont J.E. (1991). The TSH cyclic AMP cascade in the control of thyroid cell proliferation: The story of a concept. Thyroidology.

[76] Verrier B., Fayet G., Lissitzky S. (1974). Thyrotropin-binding properties of isolated thyroid cells and their purified plasma membranes. Relation of thyrotropin-specific binding to adenylate-cyclase activation. Eur. J. Biochem..

[77] Winkler F., Kleinau G., Tarnow P., Rediger A., Grohmann L., Gaetjens I., Krause G., L’Allemand D., Gruters A., Krude H. (2010). A new phenotype of nongoitrous and nonautoimmune hyperthyroidism caused by a heterozygous thyrotropin receptor mutation in transmembrane helix 6. J. Clin. Endocr Metab..

[78] Huber G.K., Weinstein S.P., Graves P.N., Davies T.F. (1992). The positive regulation of human thyrotropin (TSH) receptor messenger ribonucleic acid by recombinant human TSH is at the intranuclear level. Endocrinology.

[79] Kosugi S., Okajima F., Ban T., Hidaka A., Shenker A., Kohn L.D. (1993). Substitutions of different regions of the third cytoplasmic loop of the thyrotropin (TSH) receptor have selective effects on constitutive, TSH-, and TSH receptor autoantibody-stimulated phosphoinositide and 3′,5′-cyclic adenosine monophosphate signal generation. Mol. Endocrinol..

[80] Kosugi S., Kohn L.D., Akamizu T., Mori T. (1994). The middle portion in the second cytoplasmic loop of the thyrotropin receptor plays a crucial role in adenylate cyclase activation. Mol. Endocrinol.

[81] Kosugi S., Shenker A., Mori T. (1994). Constitutive activation of cyclic AMP but not phosphatidylinositol signaling caused by four mutations in the 6th transmembrane helix of the human thyrotropin receptor. Febs Lett..

[82] Morshed S.A., Latif R., Davies T.F. (2009). Characterization of thyrotropin receptor antibody-induced signaling cascades. Endocrinology.

[83] Gilman A.G., Rall T.W. (1968). The role of adenosine 3′,5′-phosphate in mediating effects of thyroid-stimulating hormone on carbohydrate metabolism of bovine thyroid slices. J. Biol. Chem..

[84] Rapoport B. (1976). Dog thyroid cells in monolayer tissue culture: Adenosine 3′, 5′-cyclic monophosphate response to thyrotropic hormone. Endocrinology.

[85] Allgeier A., Laugwitz K.-L., Sande J.V., Schultz G.n., Dumont J.E. (1997). Multiple G-protein coupling of the dog thyrotropin receptor. Mol. Cell. Endocrinol..

[86] Dremier S., Pohl V., Poteet-Smith C., Roger P.P., Corbin J., Doskeland S.O., Dumont J.E., Maenhaut C. (1997). Activation of cyclic AMP-dependent kinase is required but may not be sufficient to mimic cyclic AMP-dependent DNA synthesis and thyroglobulin expression in dog thyroid. Mol. Cell Biol..

[87] Kosugi S., Mori T. (1994). The first cytoplasmic loop of the thyrotropin receptor is important for phosphoinositide signaling but not for agonist-induced adenylate cyclase activation. Febs. Lett..

[88] Kosugi S., Mori T. (1994). The intracellular region adjacent to plasma membrane (residues 684-692) of the thyrotropin receptor is important for phosphoinositide signaling but not for agonist-induced adenylate cyclase activation. Biochem. Biophys. Res. Commun..

[89] Laurent E., Mockel J., Sande J.V., Graff I., Dumont J.E. (1987). Dual activation by thyrotropin of the phospholipase C and cyclic AMP cascades in human thyroid. Mol. Cell Endocrinol..

[90] Karponis D., Ananth S. (2017). The role of thyrostimulin and its potential clinical significance. Endocr. Regul..

[91] Nakabayashi K., Matsumi H., Bhalla A., Bae J., Mosselman S., Hsu S.Y., Hsueh A.J.W. (2002). Thyrostimulin, a heterodimer of two new human glycoprotein hormone subunits, activates the thyroid-stimulating hormone receptor. J. Clin. Investig..

[92] Okajima Y., Nagasaki H., Suzuki C., Suga H., Ozaki N., Arima H., Hamada Y., Civelli O., Oiso Y. (2008). Biochemical roles of the oligosaccharide chains in thyrostimulin, a heterodimeric hormone of glycoprotein hormone subunits alpha2 (GPA2) and beta5 (GPB5). Regul. Pept..

[93] Okada S.L., Ellsworth J.L., Durnam D.M., Haugen H.S., Holloway J.L., Kelley M.L., Lewis K.E., Ren H., Sheppard P.O., Storey H.M. (2006). A Glycoprotein Hormone Expressed in Corticotrophs Exhibits Unique Binding Properties on Thyroid-Stimulating Hormone Receptor. Mol. Endocrinol..

[94] Hegedüs L., Hansen J.M., Karstrup S. (1983). High incidence of normal thyroid gland volume in patients with Graves’ disease. Clin. Endocrinol..

[95] Beck-Peccoz P., Persani L., Mannavola D., Campi I. (2009). Pituitary tumours: TSH-secreting adenomas. Best Pr. Res. Clin. Endocrinol Metab.

[96] Nieto H., Boelaert K. (2016). WOMEN IN CANCER THEMATIC REVIEW: Thyroid-stimulating hormone in thyroid cancer: Does it matter?. Endocr. Relat. Cancer.

[97] Franco A.T., Malaguarnera R., Refetoff S., Liao X.H., Lundsmith E., Kimura S., Pritchard C., Marais R., Davies T.F., Weinstein L.S. (2011). Thyrotrophin receptor signaling dependence of Braf-induced thyroid tumor initiation in mice. Proc. Natl. Acad. Sci. USA.

[98] Kim Y.H., Choi Y.W., Han J.H., Lee J., Soh E.Y., Park S.H., Kim J.H., Park T.J. (2014). TSH signaling overcomes B-RafV600E-induced senescence in papillary thyroid carcinogenesis through regulation of DUSP6. Neoplasia.

[99] Moulana F.I., Priyani A.A.H., de Silva M.V.C., Dassanayake R.S. (2018). BRAF-Oncogene-Induced Senescence and the Role of Thyroid-Stimulating Hormone Signaling in the Progression of Papillary Thyroid Carcinoma. Horm. Cancer.

[100] Orim F., Bychkov A., Shimamura M., Nakashima M., Ito M., Matsuse M., Kurashige T., Suzuki K., Saenko V., Nagayama Y. (2014). Thyrotropin Signaling Confers More Aggressive Features with Higher Genomic Instability on BRAFV600E-Induced Thyroid Tumors in a Mouse Model. Thyroid.

[101] Lu C., Zhao L., Ying H., Willingham M.C., Cheng S.-y. (2010). Growth Activation Alone Is Not Sufficient to Cause Metastatic Thyroid Cancer in a Mouse Model of Follicular Thyroid Carcinoma. Endocrinology.

[102] Fournes B., Monier R., Michiels F., Milgrom E., Misrahi M., Feunteun J. (1998). Oncogenic potential of a mutant human thyrotropin receptor expressed in FRTL-5 cells. Oncogene.

[103] Rowe C.W., Paul J.W., Gedye C., Tolosa J.M., Bendinelli C., McGrath S., Smith R. (2017). Targeting the TSH receptor in thyroid cancer. Endocr Relat Cancer.

[104] Wang T., Xu J., Bo T., Zhou X., Jiang X., Gao L., Zhao J. (2013). Decreased fasting blood glucose is associated with impaired hepatic glucose production in thyroid-stimulating hormone receptor knockout mice. Endocr. J..

[105] Yan F., Wang Q., Lu M., Chen W., Song Y., Jing F., Guan Y., Wang L., Lin Y., Bo T. (2014). Thyrotropin increases hepatic triglyceride content through upregulation of SREBP-1c activity. J. Hepatol..

[106] Zhang X., Song Y., Feng M., Zhou X., Lu Y., Gao L., Yu C., Jiang X., Zhao J. (2015). Thyroid-stimulating hormone decreases HMG-CoA reductase phosphorylation via AMP-activated protein kinase in the liver. J. Lipid. Res..

[107] Song Y., Xu C., Shao S., Liu J., Xing W., Xu J., Qin C., Li C., Hu B., Yi S. (2015). Thyroid-stimulating hormone regulates hepatic bile acid homeostasis via SREBP-2/HNF-4alpha/CYP7A1 axis. J. Hepatol..

[108] Song Y., Zheng D., Zhao M., Qin Y., Wang T., Xing W., Gao L., Zhao J. (2015). Thyroid-Stimulating Hormone Increases HNF-4alpha Phosphorylation via cAMP/PKA Pathway in the Liver. Sci. Rep..

[109] Niu S., Li H., Chen W., Zhao J., Gao L., Bo T. (2018). Beta-Arrestin 1 Mediates Liver Thyrotropin Regulation of Cholesterol Conversion Metabolism via the Akt-Dependent Pathway. Int. J. Endocrinol..

[110] Zhou L., Wu K., Zhang L., Gao L., Chen S. (2018). Liver-specific deletion of TSHR inhibits hepatic lipid accumulation in mice. Biochem. Biophys. Res. Commun..

[111] Mato J.M., Alonso C., Noureddin M., Lu S.C. (2019). Biomarkers and subtypes of deranged lipid metabolism in non- alcoholic fatty liver disease. World. J. Gastroenterol..

[112] Lin Y.H., Lin K.H., Yeh C.T. (2020). Thyroid Hormone in Hepatocellular Carcinoma: Cancer Risk, Growth Regulation, and Anticancer Drug Resistance. Front. Med..

[113] Pinter M., Haupt L., Hucke F., Bota S., Bucsics T., Trauner M., Peck-Radosavljevic M., Sieghart W. (2017). The impact of thyroid hormones on patients with hepatocellular carcinoma. PLoS ONE.

[114] Chu Y.D., Lin K.H., Huang Y.H., Lin C.C., Hung C.F., Yeh T.S., Lee W.C., Yeh C.T. (2018). A novel thyroid function index associated with opposite therapeutic outcomes in advanced hepatocellular carcinoma patients receiving chemotherapy or sorafenib. Asia Pac. J. Clin. Oncol..

[115] Chi H.C., Tsai C.Y., Tsai M.M., Yeh C.T., Lin K.H. (2019). Molecular functions and clinical impact of thyroid hormone-triggered autophagy in liver-related diseases. J. Biomed. Sci..

[116] Krashin E., Piekielko-Witkowska A., Ellis M., Ashur-Fabian O. (2019). Thyroid Hormones and Cancer: A Comprehensive Review of Preclinical and Clinical Studies. Front. Endocrinol..

[117] Schottenfeld D., Gershman S.T. (1978). Epidemiology of thyroid cancer. Ca. Cancer J. Clin..

[118] Reverter J.L., Lucas A., Salinas I., Audi L., Foz M., Sanmarti A. (1992). Suppressive therapy with levothyroxine for solitary thyroid nodules. Clin. Endocrinol..

[119] Hurley J.R. (2011). Historical note: TSH suppression for thyroid cancer. Thyroid.

[120] McGriff N.J., Csako G., Gourgiotis L., Guthrie L.C., Pucino F., Sarlis N.J. (2002). Effects of thyroid hormone suppression therapy on adverse clinical outcomes in thyroid cancer. Ann. Med..

[121] Mazzaferri E.L., Jhiang S.M. (1994). Long-term impact of initial surgical and medical therapy on papillary and follicular thyroid cancer. Am. J. Med..

[122] Yoon B.H., Lee Y., Oh H.J., Kim S.H., Lee Y.K. (2019). Influence of Thyroid-stimulating Hormone Suppression Therapy on Bone Mineral Density in Patients with Differentiated Thyroid Cancer: A Meta-analysis. J. Bone Metab..

[123] Suh B., Shin D.W., Park Y., Lim H., Yun J.M., Song S.O., Park J.H., Cho B., Guallar E. (2019). Increased cardiovascular risk in thyroid cancer patients taking levothyroxine: A nationwide cohort study in Korea. Eur. J. Endocrinol..

[124] Edmonds C.J., Hayes S., Kermode J.C., Thompson B.D. (1977). Measurement of serum TSH and thyroid hormones in the management of treatment of thyroid carcinoma with radioiodine. Br. J. Radiol..

[125] Fallahi B., Beiki D., Takavar A., Fard-Esfahani A., Gilani K.A., Saghari M., Eftekhari M. (2012). Low versus high radioiodine dose in postoperative ablation of residual thyroid tissue in patients with differentiated thyroid carcinoma: A large randomized clinical trial. Nucl. Med. Commun..

[126] Paolino D., Cosco D., Gaspari M., Celano M., Wolfram J., Voce P., Puxeddu E., Filetti S., Celia C., Ferrari M. (2014). Targeting the thyroid gland with thyroid-stimulating hormone (TSH)-nanoliposomes. Biomaterials.

[127] Gao X.-j., Li A.-q., Zhang X., Liu P., Wang J.-R., Cai X. (2015). Thyroid-stimulating hormone (TSH)-armed polymer–lipid nanoparticles for the targeted delivery of cisplatin in thyroid cancers: Therapeutic efficacy evaluation. Rsc. Adv..

[128] Neumann S., Gershengorn M.C. (2011). Small molecule TSHR agonists and antagonists. Ann. Endocrinol..

[129] Neumann S., Pope A., Geras-Raaka E., Raaka B.M., Bahn R.S., Gershengorn M.C. (2012). A drug-like antagonist inhibits thyrotropin receptor-mediated stimulation of cAMP production in Graves’ orbital fibroblasts. Thyroid.

[130] Neumann S., Eliseeva E., McCoy J.G., Napolitano G., Giuliani C., Monaco F., Huang W., Gershengorn M.C. (2011). A new small-molecule antagonist inhibits Graves’ disease antibody activation of the TSH receptor. J. Clin. Endocr. Metab..

[131] Marcinkowski P., Hoyer I., Specker E., Furkert J., Rutz C., Neuenschwander M., Sobottka S., Sun H., Nazare M., Berchner-Pfannschmidt U. (2019). A New Highly Thyrotropin Receptor-Selective Small-Molecule Antagonist with Potential for the Treatment of Graves’ Orbitopathy. Thyroid.

